# High-throughput screening of ALS patient iPSC-derived spinal motor neurons identifies novel compounds that increase neurofilament light chain expression

**DOI:** 10.1016/j.slasd.2026.100303

**Published:** 2026-03-14

**Authors:** Gulcan Semra Sahin, Paul J. Guyett, Kaiping Xu, Jennifer Kouznetsova, Wei Zheng, Michael Hendrickson

**Affiliations:** a BrainXell, Inc., 455 Science Drive Suite 210, Madison, WI 53711, USA; b National Center for Advancing Translational Sciences, National Institutes of Health, 9800 Medical Center Drive, Rockville, MD 20850, USA

**Keywords:** Amyotrophic lateral sclerosis, Induced pluripotent stem cells, motor neurons, quantitative high throughput screen, Drug discovery, Drug metabolism and pharmacokinetics

## Abstract

Amyotrophic lateral sclerosis (ALS) is a fatal neurodegenerative disease primarily affecting motor neurons both in the spinal cord and brain. The cardinal pathology of ALS is motor neuron-selective inclusion of proteins such as TDP43, SOD1, C9orf72-derived dipeptide repeats, or FUS due to the mutations in the genes encoding them. Both familial and sporadic forms of ALS also show neurofilament (NF) aggregates, attributed to an imbalance in subunit expression, particularly a decrease in neurofilament light chain (NF-L) levels. Current FDA-approved treatments extend survival for only a few months, highlighting the urgent need for new therapies. In this study, we developed a cell-based reporter system for high-throughput screening by engineering induced pluripotent stem cells (iPSCs) derived from ALS patients and differentiating them into spinal motor neurons. We screened over 6000 compounds using these reporter iPSC-derived motor neurons and identified a novel compound that increases NF-L expression by >50 %. However, this novel compound also inhibits TGF-β signaling, prompting us to optimize its activity through a hit-to-lead chemistry analysis. In our subsequent investigations, we identified an additional compound that does not affect TGF-β signaling and outperforms the original compound in both *in vitro* and *in vivo* drug metabolism and pharmacokinetics assays. Our study highlights the utility of iPSC-derived neurons in disease modeling and illustrates how they can be employed to discover new compounds for therapeutic development through extensive screening in disease-relevant settings.

## 1. Introduction

Amyotrophic lateral sclerosis (ALS), also known as Lou Gehrig’s disease, is a devastating neurodegenerative disease characterized by progressive denervation of voluntary muscles due to loss of upper motor neurons in the motor cortex and lower motor neurons in the brainstem and spinal cord [1,2]. Prevalence of ALS is 6 to 9 per 100,000 persons globally with a lifetime risk of about 1 in 350 [3]. Approximately 90 % of ALS patients have sporadic ALS, with no family history of ALS-related mutations. Familial ALS, accounting for 10 % of all ALS cases, is mostly linked to mutations in genes such as *SOD1, TARDBP, FUS, C9orf72, and FUS* [4–11], causing motor neuron-selective protein inclusions of SOD1, TDP43, C9orf72 dipeptide repeat proteins, and FUS, respectively [12]. The exact mechanistic impact of these inclusions is still debated, but the final stage of disease is axonal degeneration and motor neuron (MN) death [12–15].

The predominantly sporadic nature of ALS complicates the development of targeted therapeutics. Many studies focus on various cellular aspects of the disease, such as the role of oxidative stress, mitochondrial dysfunction, and neuroinflammation to develop effective treatments [16–19]. Over 60 compounds with various mechanisms of action have been assessed in clinical trials for ALS. Currently, there are four available FDA-approved drugs to treat ALS or its symptoms: three pharmacological agents, edavarone, riluzole, and nuedexta; and one gene therapy, tofersen, for a rare form of ALS [20,21]. These drugs, and one genetic therapy silencing the *SOD1* gene [22], only modestly slow disease progression and do not provide a cure [23]. In addition, they target a small subpopulation of individuals with ALS [24]. This fact underscores the need for novel drug discoveries, either as standalone treatments or in combination with existing treatments, to enhance ALS management.

The presence of neurofilament light chain (NF-L) protein both in the cerebrospinal fluid (CSF) and in the plasma have emerged as a diagnostic biomarker of ALS [25,26]. Neurofilaments (NFs) are the principal intermediate filaments in mature neurons, and they are especially prevalent in large, myelinated axons, such as those in MNs, and MN death causes release of NF-L proteins to the CSF and plasma.

Functional NFs in the CNS are assembled through stoichiometric copolymerization of four subunits; α-internexin, the light chain (NF-L, encoded by *NEFL*), medium chain (NF-M, encoded by *NEFM*), and heavy chain (NF-H, encoded by *NEFH*) [27,28]. Intracellular NF aggregations, often due to abnormal structural organization, are a common pathological hallmark of ALS [15,29–32]. In both sporadic and familial ALS patients, *in situ* hybridization and single-cell RNA-sequencing analyses of MNs revealed a 60 % reduction in *NEFL* mRNA, but not in *NEFM* or *NEFH* mRNA [33–35]. In addition, the stability of *NEFL* mRNA is regulated by several proteins that have been linked to the pathogenesis of ALS, including SOD1, TDP-43 and RGNEF [36–38]. Transgenic expression of disease-linked mutations in model organisms results in a similar pathological outcome and this is instrumental to the understanding of disease pathogenesis and development of therapeutics [39–46]. In ALS mouse models (SOD1-G93A and SOD1-G37R), the NF-L level is also decreased in MNs, even prior to clinical disease onset [35,47]. Indeed, mating of *SOD1* mutant mice with transgenic mice overexpressing human NF-L by 1.5- to 2-fold mitigates symptoms and extends the mean longevity by 2~6 months, which represents an increase up to 65 % of their lifespan [48,49]. Paradoxically, high overexpression of NF-L in normal mice provokes an early-onset ALS-like pathology [50,51], suggesting that altered NF subunit abundance may result in incorrect NF polymerization and eventually aggregation [32].

Strikingly, NF-L reduction was also observed in MNs that are differentiated from ALS patient induced pluripotent stem cells (iPSCs) that carry mutations in *SOD1*(52) and *TARDBP* [53]. Importantly, in ALS MNs, conditional expression of NF-L back to wildtype levels restores the proper proportion of NF subunits, which correlated with decreased NF aggregation and less neurite degeneration even in the presence of an SOD1 mutation [52]. These results indicate that NF-L level reduction is a critical cause of NF aggregation, which results in a cascade of axonal degeneration and MN death. iPSC-derived MNs from ALS patients offer significant advantages for drug discovery [54]. They not only replicate the disease’s cellular environment, aiding in the understanding of disease mechanisms and progression, but their ability to be produced consistently and on a large scale also enables high-throughput screening (HTS) for new drug candidates. In this study, we generated and produced ALS patient iPSC-derived reporter spinal motor neurons (SMNs) after engineering an NanoLuc^®^ Luciferase (Nluc) reporter gene at the endogenous *NEFL* locus in the iPSCs. We performed luminescence-based HTS for >6000 compounds at multiple concentrations. Following extensive secondary analyses, we confirmed that one compound increases NF-L protein levels by >50 %. Based on structure activity relationship (SAR) analysis, we identified additional compounds and characterized them extensively through *in vitro* and *in vivo* drug metabolism and pharmacokinetics (DMPK) studies. This work introduces two novel compounds to the ongoing research aimed to develop ALS therapeutics and illustrates the potential of using patient iPSC-derived brain cells in HTS for CNS disease drug discovery.

## 2. Results

### 2.1. Generation of NLuc reporter spinal motor neurons from ALS model iPSCs

An iPSC harboring a D91A mutation (also known as D90A; dbSNP^155^ ID rs80265967) in *SOD1* was used for this work. Chen et al. reported that SMNs differentiated from the same iPSC line present ALS disease phenotype of aberrant neurofilament inclusions with reduced abundance of *NEFL* mRNA and NF-L protein in comparison to SMNs differentiated from the genetically corrected iPSCs [52]. They also demonstrated that this decrease is due to the destabilization of *NF-L* mRNA by direct binding of mutant SOD1(52).

**Fig. 1.**
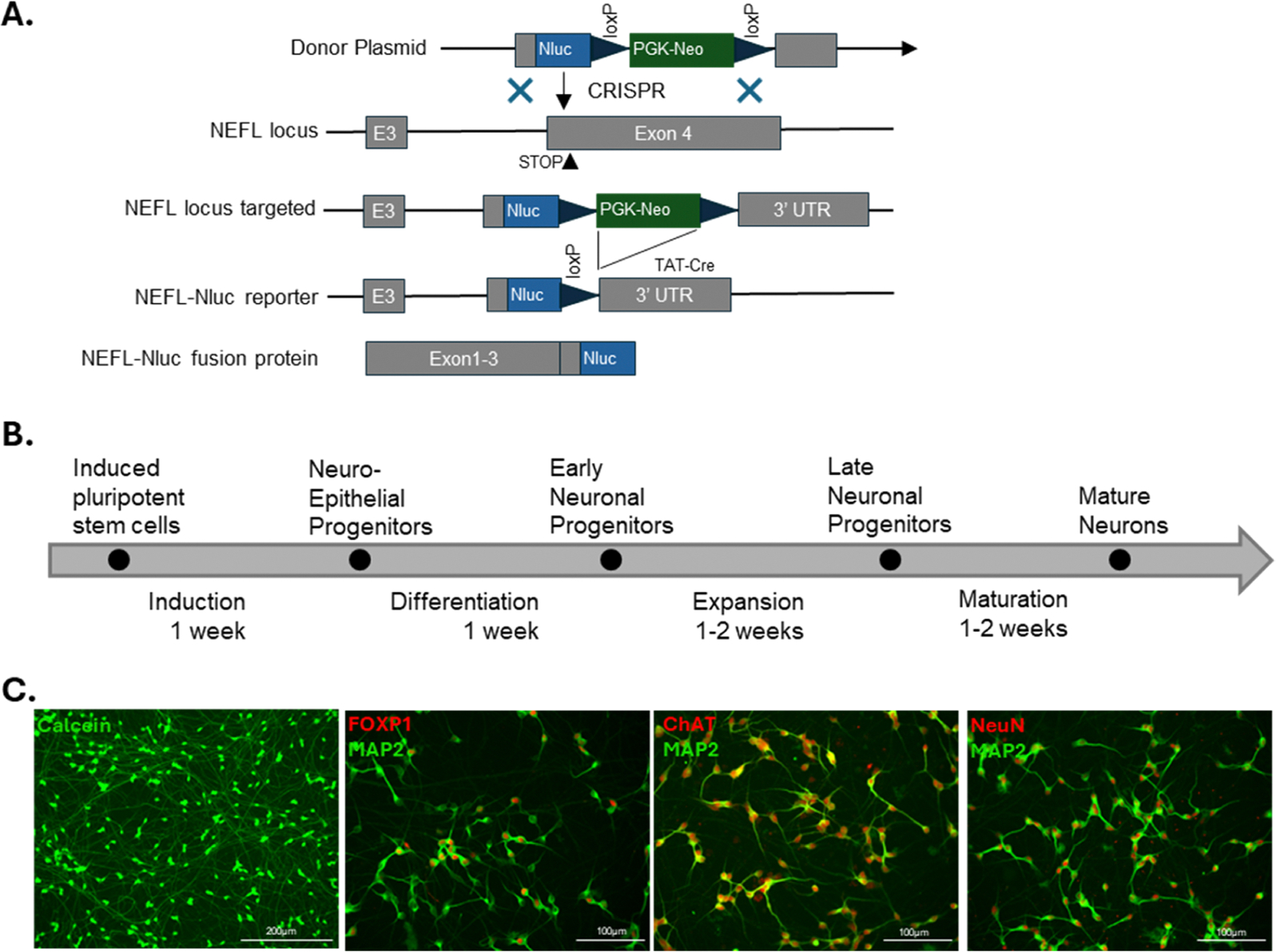
**A.** Endogenous tagging of *NEFL* gene in iPSCs to generate NF-L-NLuc reporter using CRISPR. **B.** The time from initiation of iPSC culture until cryopreservation was 4–5 weeks. Thawed and plated motor neurons mature with BrainXell maturation supplements in approximately one week. **C.** Cryopreserved neurons show high viability and express markers associated with spinal motor neuron identity, including MAP2 (>90 %), FOXP1 (>70 %), and ChAT (>85 %), NeuN (>90 %) after a week.

As we aimed to identify the compounds that could upregulate NF-L expression, we targeted the reporter gene, in this case NanoLuciferase (NLuc) [55], to one *NEFL* allele location where NLuc activity would be directly related to the endogenous transcriptional and post-transcriptional regulation of *NEFL* gene. We inserted NLuc reporter gene along with Neomycin resistant gene (*Neo*^*r*^) at the 3′ end, before the stop codon of *NEFL* gene in the iPSCs using CRISPR (Fig. 1A). By taking advantage of neomycin resistant gene, we selected successfully edited clones, and, after selection, we excised the *Neo*^*r*^ flanked by two loxP sites using a cell permeant TAT-Cre recombinase [56] (Fig. 1A). We confirmed successful insertion of NLuc gene at only one *NEFL* locus by DNA sequencing.

We differentiated the NF-L-NLuc iPSCs into SMNs following previously established differentiation protocols [52,57]. We froze the SMNs at the end of differentiation and one-week post thaw, we determined SMN survival and viability using Calcein AM cell-permeant dye, and stained to detect expression markers of SMNs, MAP2, FOXP1, ChAT and NeuN (Fig. 1B and C). We validated the successful differentiation and maturation of NLuc reporter SMN by showing that over 90 % of the cells expressed the neuron-specific marker MAP2, and >70 % of MAP2-positive neurons expressed the motor neuron-specific marker FOXP1.

### 2.2. Optimization of luminescence reporter assay for quantitative high-throughput screening in 1536-well plates

To our knowledge, no small molecule has been identified that increases NF-L protein expression. As a result, we were not able to include a positive control during our assay optimizations. Instead, we determined optimal seeding density, maturation and treatment duration to decrease well-to-well and inter-plate variation by referring to our previous HTS assays done with our SMNs (unpublished).

**Fig. 2.**
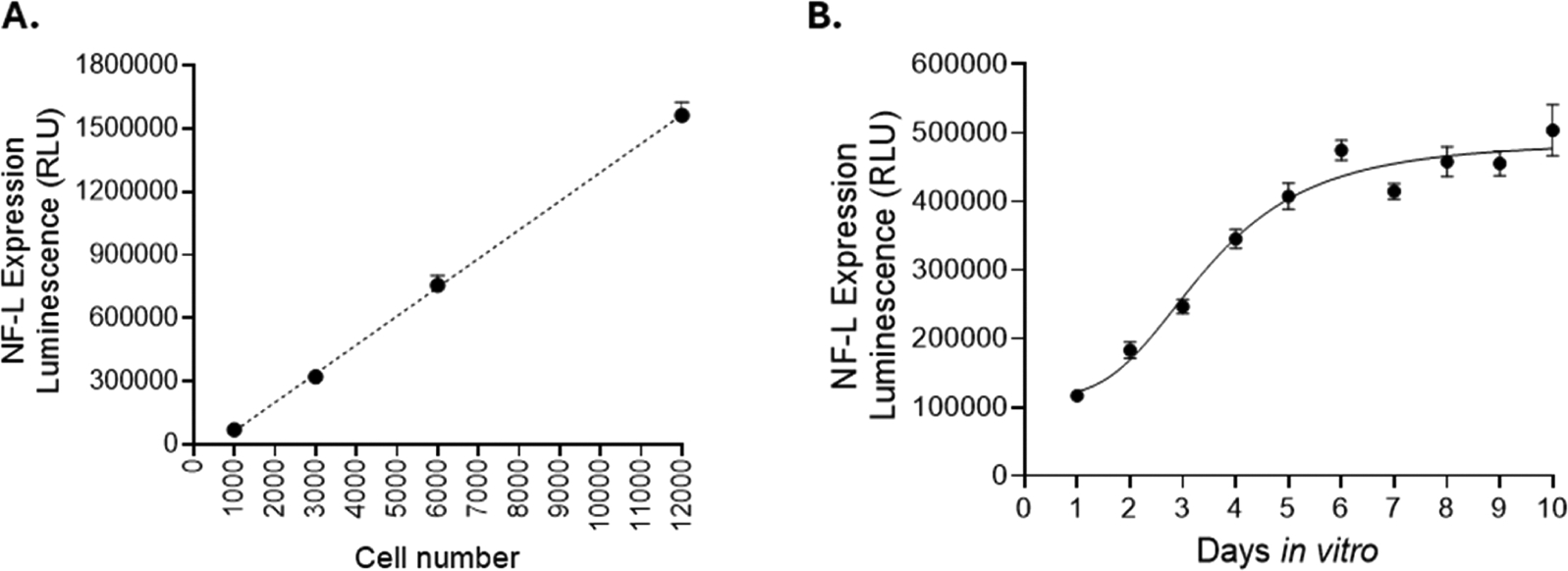
**A.** Linear increase of luminescence with the increasing number of NF-L-NLuc SMNs in 384-well plates. **B.** Time-dependent increase in NF-L expression as spinal motor neurons mature *in vitro*. A total of 4000 neurons were seeded per well in a 384-well plate. *n* ≥ 3 independent replicates for each experiment.

First, we optimized seeding density and confirmed that luminescence signal increases linearly with increasing number of SMNs, as expected (Fig. 2A). We have previously established HTS workflow using SMNs in 1536-well plates and based on this previous study (unpublished), we seeded 46,700 cells per cm^2^ of a well, or 4000 SMNs per well of a 384-well plate and 1400 SMNs per well of a 1536-well plate, which produced detectable and adequate levels of luminescence signal. In addition, we incorporated an optimized maturation supplement in our medium recipe that provided for the luminescence signal to be stabilized four days after seeding (Fig. 2B). Of note, in our previous study, we compared PDL-coated and uncoated plates for neuron seeding and observed that uncoated 1536-well plates gave better well-to-well consistency. In summary, we designed our HTS workflow to seed 1400 neurons per well; to start treatment after 24 h of seeding; and, to read NLuc activity 48 h after treatment.

### 2.3. Quantitative high-throughput screening (qHTS) of small molecule libraries

We performed HTS using three libraries: NCATS pharmaceutical collection A and B (NPC-A and NPC-B), NCATS mechanism interrogation plate (MIPE), and library of pharmacologically active compounds (LOPAC). We chose these specific libraries because they include a wide variety of compounds such as FDA-approved drugs, novel compounds entering clinical trials, and bioactive molecules targeting most signaling pathways and main drug targets.

**Fig. 3.**
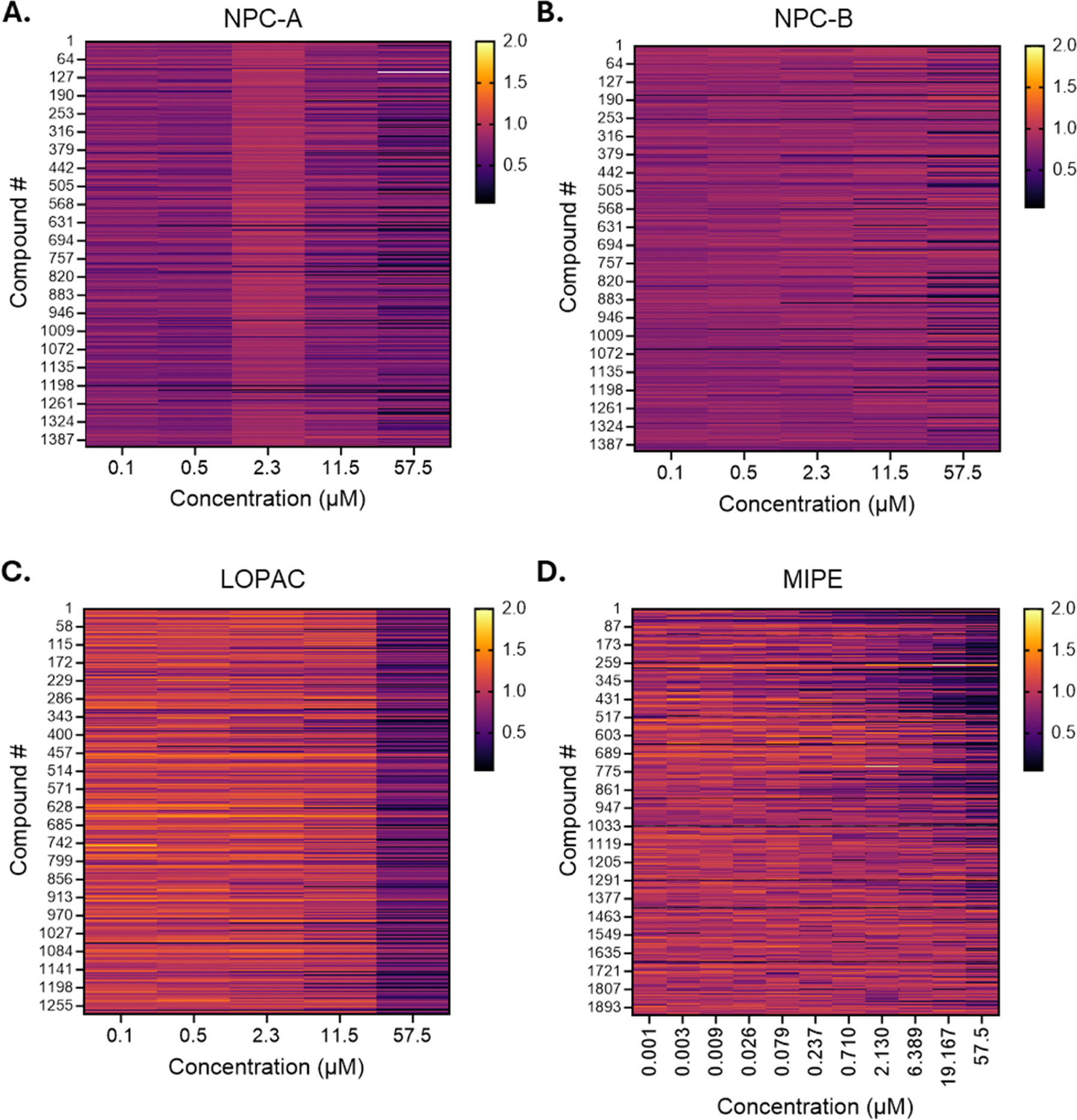
Heat map summary of luminescence signal following qHTS with **A.** NPC-A, **B.** NPC-B, **C.** LOPAC, and **D.** MIPE libraries at indicated concentrations.

The typical compound concentration for HTS assays is 1 μM. However, it is possible to miss hits when the compounds are active at higher concentrations or when they are effective at lower concentrations, but toxic at higher concentrations. Therefore, to increase rigor in hit identification, we performed 5-point dose-response qHTS: 0.1 μM, 0.5 μM, 2.3 μM, 11.5 μM, 57.5 μM for NPC-A, NPC-B, and LOPAC libraries. For the MIPE library, we performed 11-point dose-response screening: 0.001 μM, 0.003 μM, 0.009 μM, 0.026 μM, 0.079 μM, 0.237 μM, 0.71 μM, 2.13 μM, 6.39 μM, 19.17 μM, 57.5 μM. As expected, we observed increased toxicity with the majority of compounds at higher doses (Fig. 3). We normalized the NLuc signal from each compound and each concentration to the NLuc signal from vehicle conditions included in each plate. We established hit assignment threshold criteria as follows: 1) a dose-response with a positive Hill slope, 2) curve fit with R^2^ higher than 0.6, and 3) peak response greater than two standard deviations above the mean vehicle signal. Using these criteria, we identified 71 putative hit compounds for further verification (Table 1).

### 2.4. Secondary analyses of compounds identified from qHTS

More than half of our candidate hit compounds from the primary screening are known inhibitors of the various proteosome pathways and thus upregulate protein expression. We omitted these compounds from secondary analyses. We sourced independently synthesized and stored candidate compounds, which were all commercially available, for the secondary screening. This approach avoids contaminated, degraded or mislabeled compounds in HTS compound plates.

**Fig. 4.**
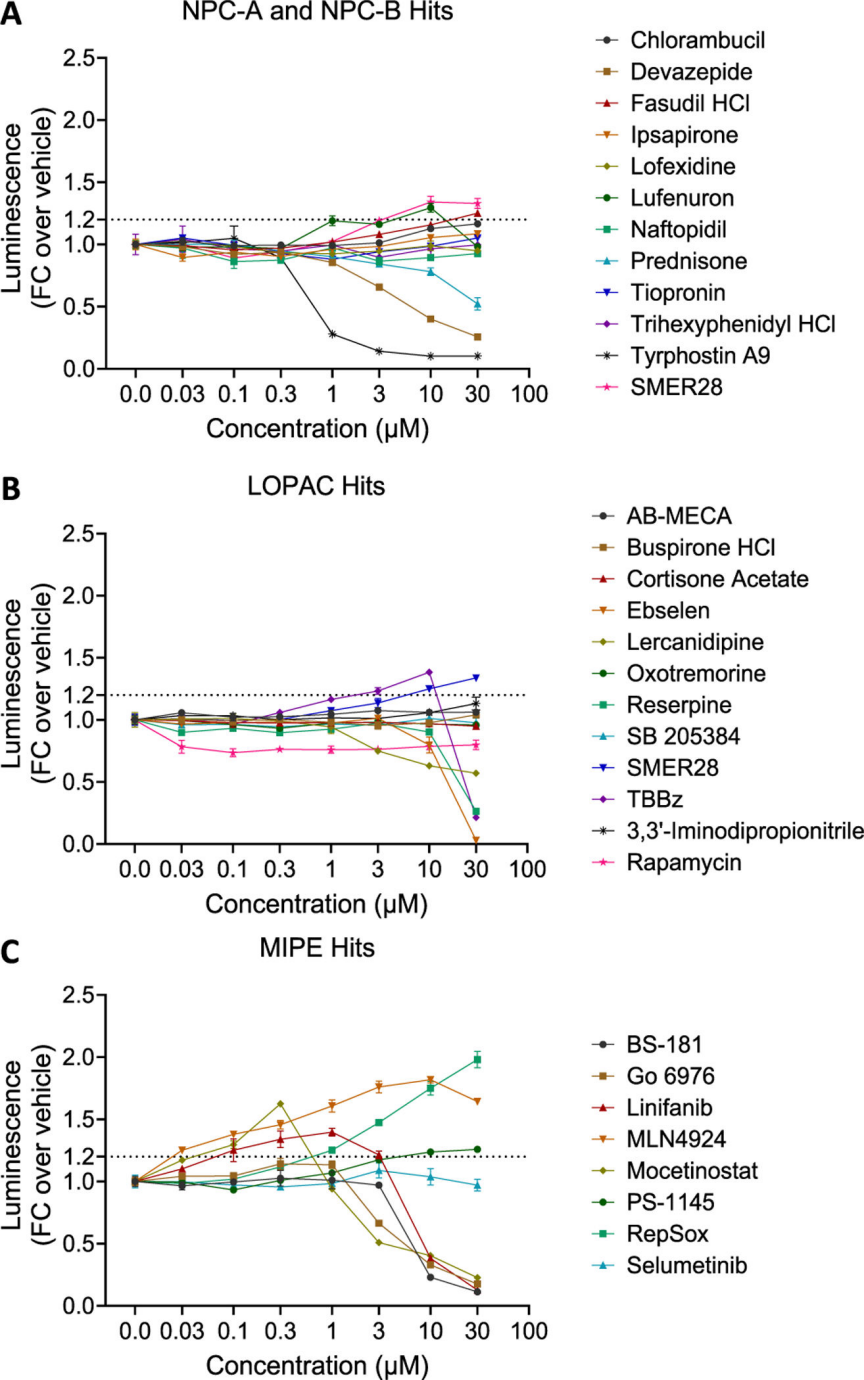
Secondary screenings of top hits from **A.** NPC-A and NPC-B, **B.** LOPAC, and **C.** MIPE libraries at various concentrations. The data represent mean ± SEM from 4 independent replicates for each concentration point. FC: Fold Change. Dotted line indicates 25 % increase over control.

**Fig. 5.**
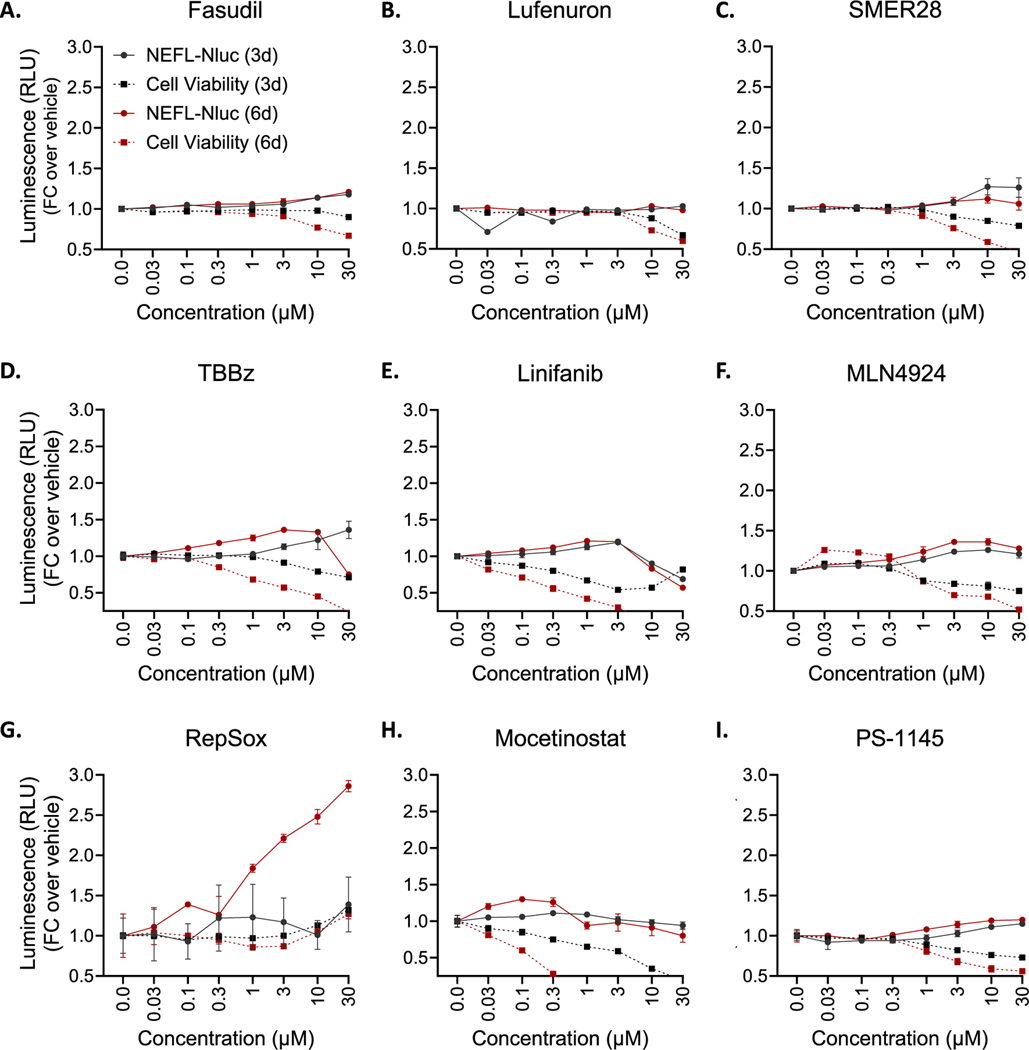
Expression of NF-L-NLuc and cell viability assessments in SOD1 D91A SMNs following treatments with compounds of interest at various concentrations at two time points. The data represent mean ± SEM from 4 independent replicates for each concentration point. FC: Fold Change.

Using a different batch of SOD1 D91A SMNs, we tested each candidate compound in 96-well plate format at 8-point concentrations; 0.03 μM, 0.1 μM, 0.3 μM, 1 μM, 3 μM, 10 μM, 30 μM, 100 μM with *n* = 4 for each concentration point. In this secondary screen, we let the SMNs mature for seven days before the treatments and measured NLuc activity three days after compound addition. Among 71 compounds, only nine compounds increased NLuc activity >20 % compared to vehicle treated neurons (Fig. 4), suggesting that the rest of the compounds were false positives. We further focused on these nine compounds and tested both three days and six days of treatment duration. We also assessed cell viability in a parallel plate. With majority of compounds, we observed increased NLuc activity when SMNs were treated for six days compared to three days at the same concentrations (Fig. 5). Interestingly, however, we observed NLuc activity to persist even with increased cell death at higher concentrations. This is most likely due to stability of NLuc enzyme in the cell culture media, released from dead cells, with a protein half-life of more than four days [58].

Among the nine tested compounds, only RepSox (E-616,452; SJN 2511) robustly increased NLuc activity with EC_50_ of 4 μM after three and six days of treatment compared to vehicle, and it did not affect cell viability (Fig. 5I).

### 2.5. RepSox increases NF-L expression and improves spinal motor neuron function independent of TGF-β receptor inhibition

**Fig. 6.**
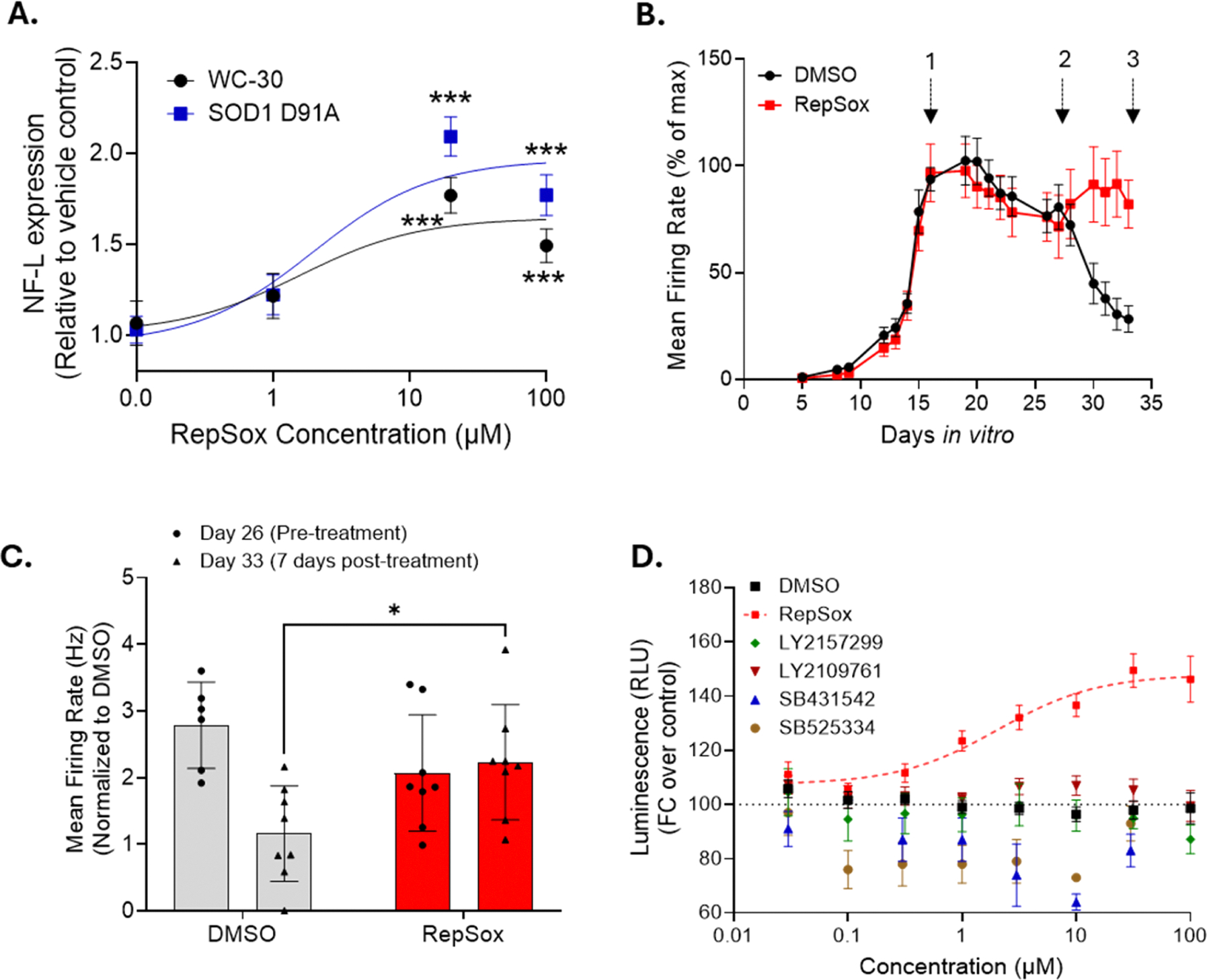
**A.** NF-L protein levels, determined with ELISA, in SMNs derived from WC-30 or SOD1 D91A hiPSC lines following treatments with indicated concentration of RepSox. **B.** Mean firing rate of SOD1 D91A SMNs for 33 days in culture. Time points to change regular culture media to minimal BrainPhys media [1], start of treatment with either DMSO or RepSox (20 μM) [2], and end of the recordings [3] are indicated. **C.** Quantification of mean firing rate of SOD1 D91A SMNs before treatment (Day 26) and 7 days after treatment (Day 33) with DMSO or RepSox. **D.** The effects of several other, highly selective, TGFβ receptor inhibitors on the NF-L expression as measured by luminescence signal. Student’s unpaired, two-tailed *t*-test (A) and two-way ANOVA, followed by Sidak’s multiple comparison test (C) are applied. The data represent mean ± SEM from ≥3 independent replicates for each experiment.

In reporter-based screens, interaction of compounds with the reporter protein rather than the desired target can yield false positives [59]. To address this issue, we measured endogenous NF-L protein levels using ELISA. This assay was done following the same protocol of seven days of maturation followed by three days of RepSox treatment in SMNs differentiated from both non-engineered, ALS patient-derived SOD1 D91A iPSCs and control WC-30 iPSCs. We confirmed that RepSox significantly increases NF-L protein levels in SMNs differentiated from both ALS patient iPSCs, SOD1 D91A and control iPSCs (Fig. 6A). Additionally, we treated two other NLuc reporter SMN lines: SMN2-NLuc, which reports on the expression levels of survival motor neuron 2 (SMN2), and CAG-NLuc, which expresses NLuc under the constitutively active CAG promoter, with varying concentrations of RepSox. However, we did not observe any upregulation of luminescence signal in either of these reporter lines (Supplementary Figure 1). These results further confirm that RepSox specifically targets *NEFL*.

It has been previously shown that NF-L is not merely a structural protein, but it is involved in regulation of synaptic transmission such that in its absence, miniature excitatory post-synaptic currents in motor neurons significantly decrease [60]. As RepSox increases NF-L expression, we wanted to test if it can also rescue ALS SMN function. To address this, we used microelectrode array (MEA) to record action potentials. As SMNs matured, the number of spikes increased each day, as expected. When we switched to minimal medium to induce stress, the number of spikes plateaued or began to slowly decrease, indicating functional motor neuron degeneration (Fig. 6B). Following incubations with RepSox or vehicle for seven days, we observed that RepSox-treated SMNs retained their functional activity, whereas vehicle-treated SMNs continued to lose their function (Fig. 6B and 6C). These data together support the disease relevant efficacy of RepSox to restore NF-L expression and improve the functional lifespan of ALS spinal motor neurons.

To determine if RepSox’s actions on NF-L expression are downstream of TGF-β-R1 inhibition and if it was a common function among other TGF-β-R1 inhibitors, we tested five additional TGF-β-R inhibitors: SB431542, SB525334, LY2109761, LY2157299, and GW788388. Interestingly, none of the compounds increased NF-L expression, even at concentrations above 30 μM (Fig. 6D). These results suggest that RepSox’s effect on NF-L expression is unique and likely occurs through activities of the compound not associated with TGF-β-R1 inhibition.

### 2.6. Compound class development through structure-activity relationship (SAR) analysis

Activation of TGF-β signaling pathways is important for survival and proliferation of many cell types in humans, and inhibitors of TGF-β-R would likely have undesirable side effects. Therefore, based on structural features of RepSox, we aimed to identify compounds that would retain RepSox’s ability to increase NF-L expression, but eliminate its inhibitor activity on TGF-β signaling. We queried all commercially available chemicals related to RepSox using the MolPort database (www.molport.com). Structure relatedness was defined by compounds that contain pyrazole and pyridine ring substructures, including fused ring systems, with molecular weights below 650 Da. We identified a total of 130 compounds with a diverse set of substitutions to each ring system of RepSox (Supplementary Figure 2).

**Fig. 7.**
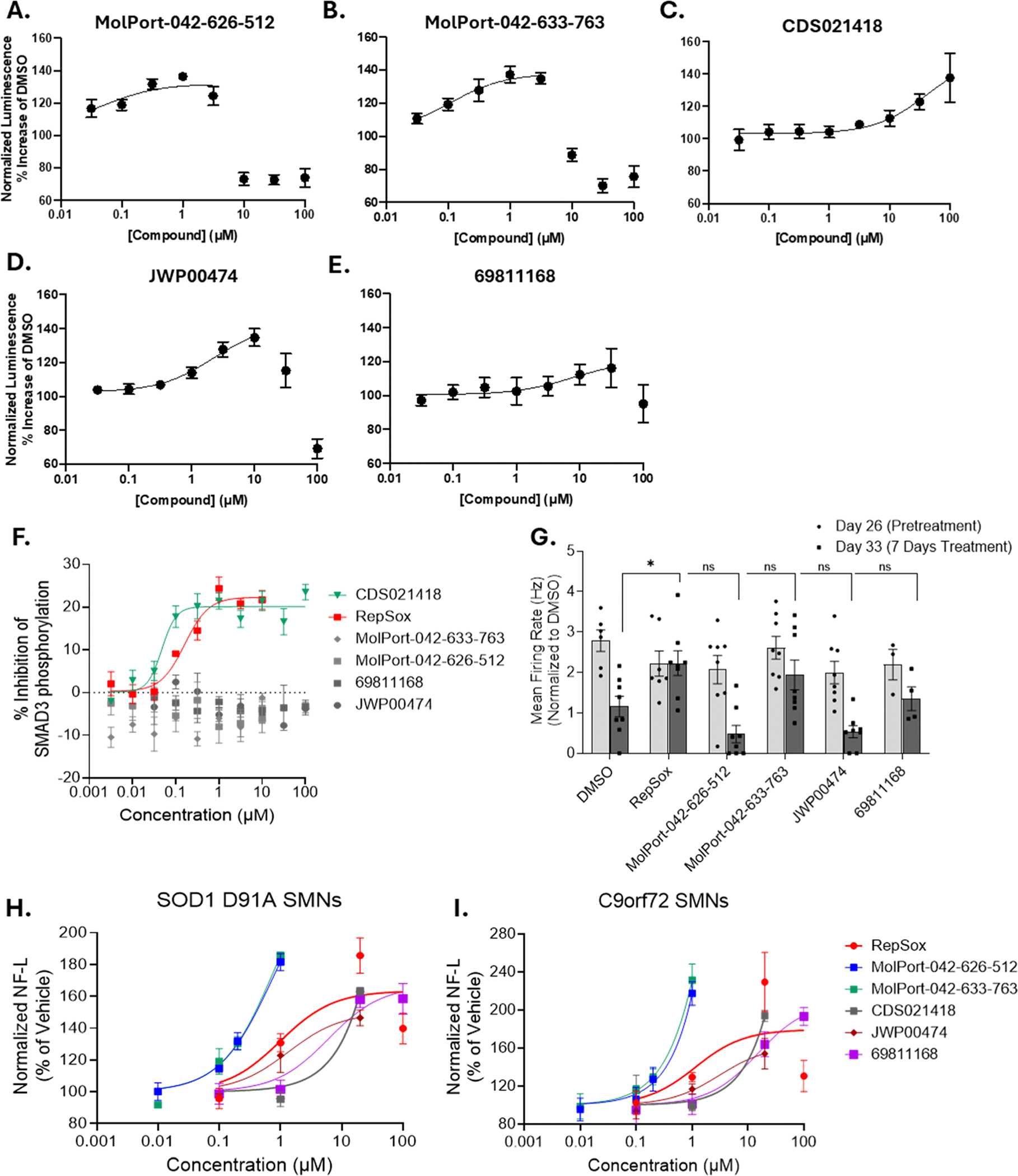
**A-E.** Dose-response curves of 5 compounds identified with SAR analysis for NF-L expression. **F.** The effect of RepSox and several structurally similar several compounds on inhibition of SMAD3 phosphorylation. **G.** Quantification of mean firing rate of SOD1 D91A SMNs before treatment (Day 26) and 7 days after treatment (Day 33) with DMSO or other candidate compounds. **H-I.** NF-L protein levels were determined with ELISA in SMNs derived from **H.** SOD1 D91A and **I.** C9orf72 iPSCs following treatments with indicated concentrations of RepSox and 5 additional compounds. Two-way ANOVA, followed by Sidak’s multiple comparison test is applied for **G**. The data represent mean ± SEM from ≥3 independent replicates for each experiment.

We screened these new compounds in 384-well plate format using NLuc reporter SOD1 D91A SMNs. Following neuron maturation in the culture for seven days, we treated them with the compounds for three days at following concentrations: 0.03 μM, 0.1 μM, 0.3 μM, 1 μM, 3 μM, 10 μM, 30 μM, and 100 μM. Substitutions at the pyrazole R1 position ablated activity, including cyclic pentane conjugation at R1-R4. Interestingly, all compounds with a 2-substitute 1,5-naphthyridine at R3 were active with similar profiles, even though these possessed various substitutions at R2, including complete removal of the pyridine. Of the 130 compounds, only five were active in the NLuc reporter assay, providing valuable insight into the structural constraints to achieve NF-L upregulation (Fig. 7A–E). These results suggest that R2 substituents contribute minimally to compound activity, whereas R3 substituents correlate strongly with NF-L activity, especially when the substituent is a naphthyridinyl fused bicycle. When we compared the activity of a series of compounds containing biarylheteroaromatic rings substituted at the pyrazole R3-position, it was noted that the key to activity is the distance and orientation of a single nitrogen with respect to the pyrazole. For example, significant activity is seen when a 1,5-naphthyridine (referring to the location of its nitrogen atoms) is attached through its 2-position to the pyrazole. Among 5 active compounds, we found that MolPort-042–626–521 and MolPort-042–633–763 have 40-fold improved potency with efficacy approaching a 40 % increase in NF-L expression (Table 2).

Next, we assessed if any of the five compounds affect the canonical TGF-β/SMAD3 signaling pathway. TGF-β-R signals through SMAD3 by phosphorylating the SSxS C-terminal domain [61,62], and so we measured SMAD3 phosphorylation levels to detect TGF-β-R activation or inhibition following various compound treatments in HepG2 cells. We found that one of the compounds, CDS021418, inhibited SMAD3 phosphorylation and excluded it from the subsequent experiments. Conversely, MolPort-042–626–521 and MolPort-042–633–763 did not affect TGF-β-R/SMAD3 signaling (Fig. 7F). We also performed the MEA assay using four compounds that did not inhibit the canonical TGF-β/SMAD3 signaling pathway. Treatment with 0.5 μM MolPort-042–633–763 extended the activity of SOD1 D91A SMNs almost as well as RepSox (Fig. 7G). Via ELISA, we confirmed that MolPort-042–633–763 upregulates NF-L protein expression both in SOD1 D91A SMNs and another ALS model, C9orf72 SMNs (Fig. 7H and I).

### 2.7. Drug metabolism and pharmacokinetics (DMPK) studies

Performing *in vivo* studies using ALS mouse models requires intensive labor and major financial commitment. To determine which compounds will advance to further testing, we first conducted a series of DMPK studies. The objective of these preliminary tests was to identify which compounds could achieve a therapeutic concentration in the CNS while maintaining a non-toxic dose level. We, therefore, analyzed the compounds in a series of *in vitro* assays to measure (i) stability in liver microsomes, (ii) plasma protein binding, and (iii) permeability to both the intestine and the blood-brain barrier. The results obtained from our studies along with DMPK studies are summarized in Table 2. Based on these results, RepSox and MolPort-042–633–763 were prioritized for *in vivo* studies.

**Fig. 8.**
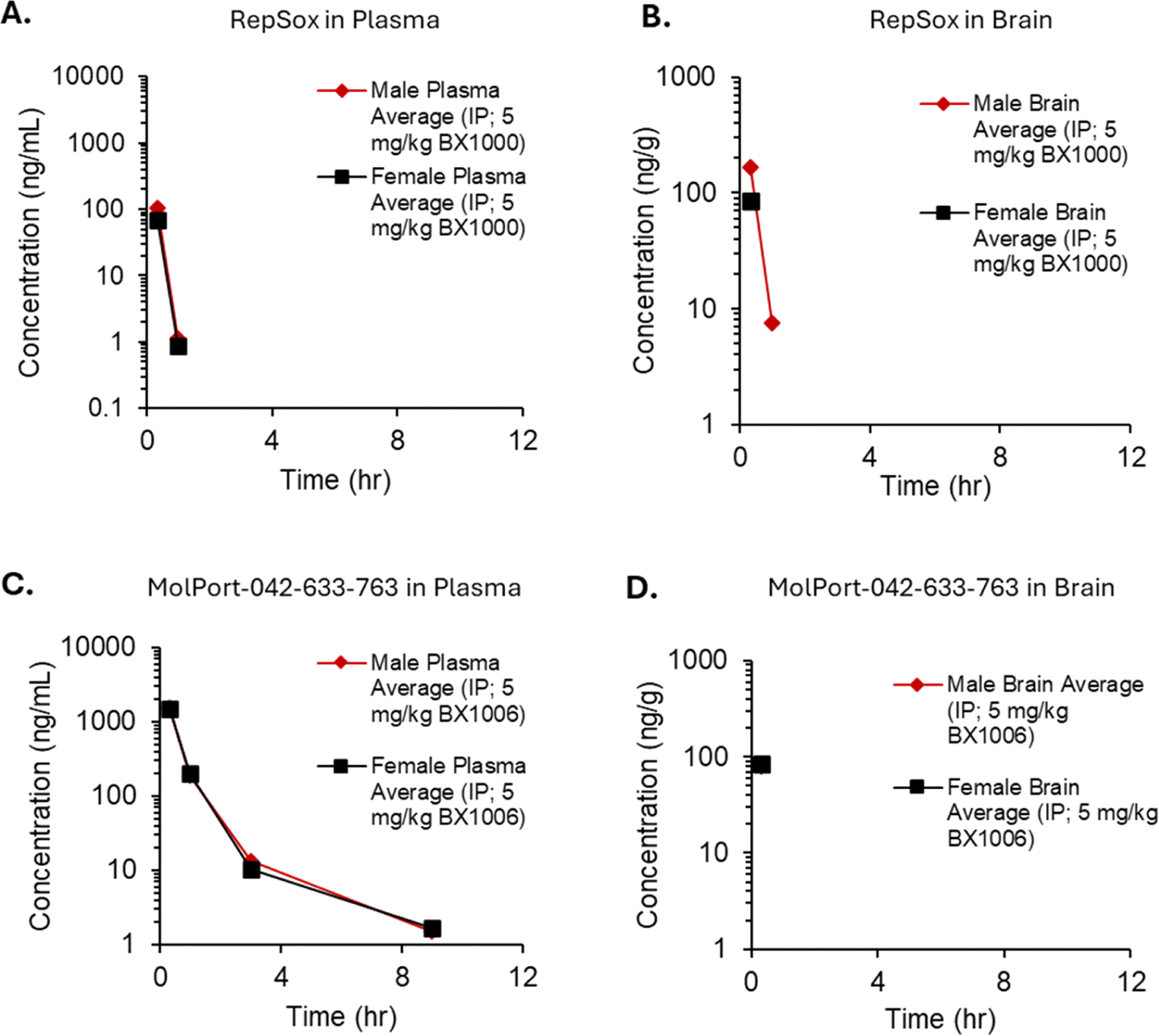
Pharmacokinetics and brain-to-plasma ratios of the RepSox and MolPort-042–633–763 in male and female CD-1 mice. **A.** Plasma and **B.** brain concentrations of RepSox after intraperitoneal (IP) administration at 5 mg/kg in mice. **C.** Plasma and **D.** brain concentrations of MolPort-042–633–763 after IP administration at 5 mg/kg in mice. BX1000: RepSox; BX-1006: MolPort-042–633–763.

*In vivo* DMPK studies evaluated brain-to-plasma ratios of RepSox and MolPort-042–633–763 both in male and female CD-1 mice following intraperitoneal (IP) administration of compounds at 5 mg/kg. No toxicity was detected in the mice throughout the study with either of the compounds. We observed that both compounds can cross the blood-brain barrier (Fig. 8C and D). MolPort-042–633–763 exhibited an enhanced plasma half-life compared to RepSox (Fig. 8A and C). However, both compounds were rapidly cleared from the brain and plasma, suggesting that further improvements in these metrics are necessary before advancing to ALS model mouse studies.

## 3. Discussion

Induced pluripotent stem cell (iPSC)-derived cells have been widely used for *in vitro* human disease modeling, drug screening, and development of cell therapies over the last two decades [63,64]. iPSC-derived neurons are especially useful in disease modeling as many neurodegenerative diseases are difficult to recapitulate in rodent models. Additionally, access to primary human brain cells in large quantities to perform drug screening is highly limited. iPSC-derived neurons provide a valuable platform for drug screening as iPSCs are amenable to genetic engineering to create a cell-based reporter system, they can be produced at large scale to support high throughput screening, and, most importantly, iPSCs can be derived from patients to produce relevant disease model platforms [65].

We successfully produced ALS patient-derived reporter iPSCs and differentiated them at large scale to motor neurons (MNs) to execute phenotypic high-throughput screening. We screened approximately 6000 compounds at either 5 or 11 concentration points as an initial step. To our knowledge, there is no known compound that can increase the expression of NF-L. Therefore, to enhance rigor and minimize false negatives—such as compounds with low EC_50_ values that induce toxicity at moderate concentrations—we chose to evaluate multiple doses instead of relying on a single dose with several technical repeats for our primary screening. We set up our screening efficacy threshold to call hits between 30–40 % since a majority of compounds did not increase NF-L levels beyond 50–60 %. For our purposes, an increase in NF-L levels by 50 % was the desired response target. It is important to emphasize here that neurofilaments are one of the primary structural components of mature neurons [66] and elevated NF-L levels in cerebrospinal fluid and plasma serve as biomarkers for neuron death in various neurodegenerative diseases, including ALS and traumatic brain injury. Our main approach is to restore, not to over-express, NF-L levels in healthy MNs before the disease progression, thereby balancing the neurofilament subunit pool to decrease neurofilament aggregation.

Following extensive analyses on candidate compounds, we identified RepSox, a TGF-β-R1 receptor inhibitor, that increased NF-L expression without causing cytotoxicity. To the best of our knowledge, this is the first study to identify a pharmacological agent that increases NF-L expression levels. However, a significant downside of RepSox activity was that it inhibits TGF-β receptor activity at much lower concentrations than it upregulates NF-L expression. To understand if NF-L expression upregulation was downstream of TGF-β signaling, we tested several other highly potent, TGF-β receptor inhibitors. To our surprise, none of them upregulated NF-L expression, suggesting that RepSox activity on NF-L expression is independent of its activity on the TGF-β-R1 receptor. This encouraged us to search for a structurally similar compound that conserves its role in NF-L expression regulation but eliminates its effect on TGF-β receptor inhibition. Structure-activity relationship analyses identified an additional 130 compounds. Although most of them were inactive as expected, five compounds increased NF-L levels similarly to RepSox. Among the five compounds, four did not activate the canonical TGF-β/SMAD3 pathway and were subsequently evaluated *in vitro* functional MEA studies. Notably, these candidate compounds may potentially activate non-canonical, SMAD3-independent signaling pathways mediated by TGF-β receptors. To gain a comprehensive understanding of affected signaling pathways and downstream targets of the candidate compounds, future studies will be crucial to conduct multi-omics studies such as proteomics, transcriptomics, and gene set enrichment analysis (GSEA) in motor neurons.

We performed extensive *in vitro* DMPK studies to choose compounds that are most suitable for pre-clinical studies. We identified two compounds to prioritize for *in vivo* preliminary studies. MolPort-042–633–763 performed better *in vitro* studies as it increases NF-L expression at significantly lower concentrations. Furthermore, the therapeutic index, defined as EC_50_(NF-L)/EC_50_(Toxicity), of MolPort-042–633–763 is significantly higher than RepSox, even though neither of the compounds reach the desired threshold of >100. Additional work is necessary to identify the exact molecular pathways through which RepSox or MolPort-042–633–763 increases NF-L expression, and it is possible that finding the molecular mechanisms would identify additional compounds that can restore NF-L expression with much less toxicity, significantly improving the therapeutic index.

Pre-clinical studies involving ALS mouse models require significant resources, both in terms of financial investment and time commitment. We therefore used CD-1 mice, a commonly utilized mice in toxicology testing, to understand the ability of the two compounds to cross the blood-brain barrier and their retention in the plasma and brain. This study was crucial to understand how structurally similar compounds differ at their plasma retention, and MolPort-042–633–763 had better plasma retention compared to RepSox. Nonetheless, both compounds were rapidly cleared from the brain and the plasma, requiring further improvements in both metrics before entering ALS mouse model studies.

In conclusion, we successfully applied patient iPSC-derived spinal motor neurons to model ALS disease and perform high throughput screening to identify a novel compound to advance therapeutics efforts against ALS. We optimized hit-to-lead chemistry via structure-activity relationship studies to identify an additional compound and demonstrated that the optimized compound displayed better performance through series of *in vitro* and *in vivo* drug metabolism and pharmacokinetics studies. We anticipate that these novel compounds will provide a novel approach to eliminate neurofilament protein aggregates that are common among all ALS forms.

## 4. Materials and methods

### 4.1. Establishment of reporter iPSC lines

The initial SOD1-D91A iPSC line was generated as described by Dr. Zhong-Wei Du and approved by the stem cell research oversight committee, University of Wisconsin-Madison [52]. Briefly, fibroblasts from a 50-year-old female carrying the D90A SOD1 mutation (ND29149, P3, Coriell Institute, http://www.coriell.org), were reprogrammed using the nonintegrating Sendai virus as described (Ban et al., 2011). The NLuc-reporter line was produced at the Waisman Center hPSC Service core facility at the University of Wisconsin-Madison. Briefly, clustered regularly interspaced short palindromic repeats (CRISPR) technology was used to integrate the NLuc reporter into the endogenous *NEFL* gene before the stop codon. CRISPR guide RNA pairs, Cas9 nickase Cas9D10A (Addgene plasmid #44,720), and a donor plasmid were introduced into the iPSCs by electroporation. Neomycin (100 ng/ml) was added in the culture medium to select resistant cells. The neomycin-resistant iPSC colonies were picked and screened by PCR to confirm the insertion of *NLuc* reporter. Two primer pairs were used to confirm the 5′ and 3′ junctions created during integration as well as the wild-type *NEFL* sequence. Clones positive in both PCR reactions were selected as they confirmed that only one *NEFL* allele was targeted.

In the donor plasmid, we flanked the neomycin resistant cassette with two loxP sites; therefore, it was removed in culture with 2 μM purified Tat-Cre protein (Excellgen, Cat. no. EG-1001). All the reporter iPSC lines were confirmed without mutation in *NEFL* gene or off-target sites. The potential off target sites were selected according to online tools provided by Feng Zhang’s lab (http://www.rgenome.net/cas-offinder/)[67].

### 4.2. Spinal motor neuron differentiation from human iPSCs

Spinal motor neuron (SMN) differentiation from human iPSCs was based on protocols described previously [68]. Briefly, human iPSCs were treated with small molecules for 1 week to induce neuroepithelial progenitors (NEPs). The NEPs were split and treated in additional patterning molecules, retinoic acid and SHH, for another week to generate subtype-specific neuron progenitors. These progenitors were expanded and further differentiated with the same combination of small molecules to generate at least 10^9^ OLIG2^+^ motor neurons per batch. The large batch yield was aliquoted and cryopreserved in freezing medium until used. After thawing, viable cells were determined by ethidium exclusion and counted using an automated counter, Countess II FL (ThermoFisher Scientific). Cells were then resuspended in culture medium (1:1 DMEM/F12 and Neurobasal medium supplemented with 1X B-27, 1X N-2, 0.25X GlutaMAX, and 15 μg/mL Geltrex) and plated as needed for each application. To accelerate maturation after plating, neurons were cultured in medium supplemented with BrainFast Motor neuronal maturation supplement (BrainXell, Cat. no. BX-2100).

### 4.3. Spinal motor neuron immunofluorescence

SMNs were plated (1 × 10^4^/well) in black clear bottom, PDL-coated 96-well PhenoPlates (Revvity, Cat. no. 6055,302). After 7 days of culture at 37 °C, 5 % CO_2_ the neurons were washed once with PBS and fixed in 4 % paraformaldehyde for 10 min. Fixed cells were washed twice, 5 min each, with PBS and blocked in blocking solution (0.1 % Triton X-100 and 2.5 % donkey serum in 1X D-PBS) for 10 min. After blocking, the primary antibodies were added in the blocking solution at the following dilutions: anti-MAP2, 1:1000 (Millipore Sigma, Cat. no. M1406); anti-NeuN, 1:1000 (Abcam, Cat. no. EPR12763); FOXP1, 1:1000 Millipore Sigma, Cat. no. ab177487); and anti-ChAT, 1:500 (Abcam, Cat. no. AB181023). The primary antibodies were incubated overnight at 4 °C. The next day, the cells were washed twice, 5 min each, with PBS and blocked in re-blocking buffer (5 % donkey serum in 1X D-PBS) for 10 min. After re-blocking, secondary antibody (1:1000 dilution of goat anti-mouse conjugated to iFluor 488 (AAT BioQuest, Cat. no. 16,773) or goat anti-rabbit conjugated to iFluor 555 (AAT BioQuest, Cat. no. 16,831) was added in re-blocking buffer supplemented with Hoechst stain (1:2000, Biotium, Cat. no. 446). Secondary antibodies and counter stain were incubated for 45 min at room temperature and then washed twice with PBS. Cells were imaged using an automated Evos FL Auto 2 microscope (Thermo Fisher Scientific). Marker staining was quantified in two microscopy fields per well for a total of 6 wells.

### 4.4. Quantitative HTS screening and verification

Motor neurons were thawed and resuspended at 3.5 × 10^5^ per mL in culture medium. Cell viability and density were confirmed (described above), and cells were plated at 1400 cells/well in 4 μL using a MultiDrop Combi (Thermo Fisher Scientific) into white uncoated 1536-well plates (and one μClear plate). On Day 1 (24 h after thawing and plating), compounds were added by an automated pin tool dispenser (Wako Automation) in 23 nL DMSO at desired concentrations. On Day 3, NLuc activity was detected using the Nano-Glo Luciferase Assay kit (Promega, Cat. no. 1150) following the manufacturer protocol. Luminescence signal was measured after 15 min incubation using a ViewLux plate reader (PerkinElmer).

Plate data from LOPAC, MIPE, and NPC qHTS were archived and analyzed using Collaborative Drug Discovery (CDD) vault. Standard dose-response analysis in the CDD Vault was used for normalization, and nonlinear regression for best-fit analysis was performed.

The candidate hits from the above 1536-well assays were rescreened in the 384-well and 96-well plate format following a similar protocol. Briefly, SMNs were thawed and plated (6000 per well in 384-well PDL-coated μClear plates or 25,000 per well in 96-well PDL-coated μClear plates (Greiner Bio-One, Cat. no. 655,088) in culture medium (50 or 100 μL) and allowed to mature for two days. On Day 2, compounds were serially diluted and added to cell culture plates using a PipetMax liquid handler (Gilson) in 50 or 100 μL base medium (culture medium without BrainFast and Geltrex). On Day 4 (48 h post compound addition), the medium was removed and NLuc activity determined using Nano-Glo Luciferase assay. Plates were read using a Spark multimode microplate reader (Tecan), and the data were normalized and analyzed using nonlinear regression for best-fit analysis in GraphPad Prism 10.

### 4.5. ELISA

Spinal motor neurons were plated at 25,000 per well in 96-well PDL coated μClear plates in 100 μL culture medium. On Day 2, compounds were added in 100 μL of base medium. On Day 7, the amount of NF-L protein was analyzed using an ELISA kit (Uman Diagnostics, Cat. no. 20–8002 RUO) following manufacturer’s instructions. Absorbance (450 nm ± 3 nm) of the developed ELISA was measured using a Tecan Spark multimode microplate reader. Data were analyzed using GraphPad Prism 10, applying a nonlinear regression model for best-fit analysis.

### 4.6. Microelectrode array (MEA) analysis

SOD1 D91A SMNs were seeded at 100,000 cells per well in 24-well multielectrode array (MEA) plates (Axion BioSystems). Plates were previously coated with PDL. The SMNs were matured for 14 days with complete medium supplemented with BrainFast Motor neuronal maturation supplement. After maturation, they were transitioned to minimal medium (BrainPhys neuron culture medium) to induce a stressed condition. After 12 days in minimal medium, day 26 of *in vitro* culture, the SMNs were treated with 20 μM RepSox or equivalent amount of DMSO solvent. SMN function as number of action potentials, or “spikes” was measured over a 15 min recording at 37 °C, 5 % CO_2_ using a Maestro Edge (Axion Biosystems). The experiment was repeated eight times (two biological replicates with four technical replicates each) and the number of spikes observed each day was plotted *versus* days *in vitro*.

### 4.7. SMAD3 phosphorylation assay

An optimized high throughput compatible time-resolved fluorescence resonance energy transfer-based assay (HTRF) kit to detect total SMAD3 and phospho-SMAD3 (Ser423/425) (Revvity, Cat. no. 63ADK025PEG). The ratio of phospho-SMAD3 to total SMAD3 indicated the level of TGF-β receptor activation. The level of phospho-SMAD3 approaches 0 % when no TFG-β is present and approaches 100 % of total SMAD3 in the presence of 1 ng/mL TGF-β. The HTRF assay was optimized using HepG2 cell line.

### 4.8. Plasma protein binding

This study was performed by Absorption Systems. Briefly, assays were carried out in mixed-gender human plasma collected on sodium heparin (BioIVT, Cat. no. AS1465–88). A Pierce Rapid Equilibrium Dialysis (RED) device was used for all experiments. Stock solutions of the test articles and control compound were first prepared in DMSO. Aliquots of the DMSO solutions were spiked into 1.0 mL of plasma at a dosing concentration of 5 μM for the test article and 10 μM for the co-dosed control compound, warfarin. Plasma (300 μL), containing test article and control compound, was loaded into two wells of the 96-well dialysis plate. Phosphate-buffered saline (PBS) (500 μL) was added to each corresponding receiver chamber. The device was then placed into an enclosed heated rocker pre-warmed to 37 °C and allowed to incubate for four hours. After four hours of incubation, both sides were sampled. Aliquots (50 μL for donor, 200 μL for receiver) were removed from the chambers and placed into a 96-well plate. Plasma (50 μL) was added to the wells containing the receiver samples, and 200 μL of PBS was added to the wells containing the donor samples. Two volumes of acetonitrile (ACN) were added to each well, and the plate was mixed and then centrifuged at 3000 rpm for 10 min. Aliquots of the supernatant were removed, diluted 1:1 into water, and analyzed by LC-MS/MS.

Protein binding values were calculated as follows:

%Bound=[(PARRinDonor−PARRinReceiver)/(PARRinDonor)]×100

PARR = peak area response ratio to internal standard, including applicable dilution factors.

### 4.9. Stability in liver microsomes

This study was performed by Absorption Systems using mixed-gender human liver microsomes (XenoTech, Cat. no. 1010,420). The reaction mixture, minus the cofactor, NADPH was prepared. The test article was added into the reaction mixture at a final concentration of 1 μM. The control compound, testosterone, was run simultaneously with the test article in a separate reaction. An aliquot of the reaction mixture (without cofactor) was equilibrated in a shaking water bath at 37 °C for three minutes. The reaction was initiated by the addition of the cofactor, and the mixture was incubated in a shaking water bath at 37 °C. Aliquots (100 μL) were withdrawn at 0, 10, 20, 30, and 60 min after cofactor addition. Test article and testosterone samples were immediately combined with 400 μL of ice-cold 50/50 acetonitrile (ACN)/H2O containing 0.1 % formic acid and internal standard to terminate the reaction. The samples were then mixed and centrifuged to precipitate proteins. All samples were assayed by LC-MS/MS using electrospray ionization. The peak area response ratio (PARR) to internal standard was compared to the PARR at time 0 to determine the percentage remaining at each time point. Half-lives and clearance were calculated using GraphPad software, fitting to a single-phase exponential decay equation.

### 4.10. Bidirectional permeability in Caco-2 monolayers

This study was performed by Absorption Systems. Caco-2 cells (ATCC, clone C2BBe1) were grown as monolayers to confluence on collagen-coated, microporous membranes in 12-well assay plates. The permeability assay buffer was Hanks’ balanced salt solution (HBSS) containing 10 mM HEPES and 15 mM glucose at a pH of 7.4. The buffer in the receiver chamber also contained 1 % bovine serum albumin (BSA). The dosing solution concentration was 5 μM of test article in the assay buffer. Cell monolayers were dosed on the apical side (A-to-B) or basolateral side (B-to-A) and incubated at 37 °C with 5 % CO_2_ in a humidified incubator. Samples were taken from the donor and receiver chambers at 120 min. Each condition was performed in duplicate. The flux of lucifer yellow was also measured post-experimentally for each monolayer to ensure no damage was inflicted to the cell monolayers during the flux period. All samples were assayed by LC-MS/MS using electrospray ionization.

The apparent permeability (Papp) and percentage recovery were calculated as follows:

(1) $$P_{\text{app}}=\left(dC_{\text{r}}/d\text{t}\right)\times {\text{V}}_{\text{r}}/\left(\text{A}\times {\text{C}}_{\text{A}}\right)$$

(2) $$\text{PercentageRecovery}=100\;\;\;\;\;\;\;\;\;\;\;\;\times \left(\left(V_{r}\times C_{r}^{\text{final}}\right)+\left(V_{d}\times C_{d}^{\text{final}}\right)\right)/\left(V_{d}\times C_{N}\right)$$

In these equations, $d{\text{C}}_{\text{r}}/d\text{t}$ is the slope of the cumulative concentration in the receiver compartment *versus* time in μM s^−1^; V_r_ is the volume of the receiver compartment in cm^3^; V_d_ is the volume of the donor compartment in cm^3^; A is the area of the insert (1.13 cm^2^ for 12-well); $C_{A}$ is the average of the nominal dosing concentration and the measured 120-minute donor concentration in μM; C_N_ is the nominal concentration of the dosing solution in μM; Cr^final^ is the cumulative receiver concentration in μM at the end of the incubation period; and $C_{\text{d}}^{\text{final}}$ is the concentration of the donor in μM at the end of the incubation period.

Efflux ratio (ER) is defined as P_app_ (B-to-A) / P_app_ (A-to-B).

### 4.11. In-vitro determination of blood-brain barrier penetration potential

This study was performed by Absorption Systems. MDR1-MDCK cell monolayers were grown to confluence on collagen-coated, microporous membranes in 12-well assay plates. This assay was conducted exactly as in the same manner as the bidirectional permeability assay in Caco-2 monolayers described above.

### 4.12. Pharmacokinetics and brain-to-plasma ratios in CD-1 mice

This study was performed by Absorption Systems. In the study, the brain-to-plasma ratios of the RepSox and Molport-042–633–763 were evaluated in male and female CD-1 mice following intraperitoneal (IP) administration at 5 mg/kg. Blood and brain samples were collected at 20 min, 1-, 3-, 9-, and 24-hours post-dose in male and female mice. Plasma and brain concentrations of the RepSox and Molport-042–633–763 were determined by LC-MS/MS, and the brain-to-plasma ratios were calculated.

## Supplementary Material

MMC1

Supplementary material associated with this article can be found, in the online version, at doi:10.1016/j.slasd.2026.100303.

## Figures and Tables

**Table 1:** Summary of qHTS data and number of hits detected from each library at indicated thresholds.

Library	Library Size	Threshold (% increase)	Hits

NPC-A	1408	30 %	22
NPC-B	1408	30 %	10
LOPAC	1280	40 %	21
MIPE	1920	30 %	18

**Table 2:** Hit compound results from *in vitro* MN assays and DMPK studies. The values that meet the threshold criteria are not highlighted. Yellow highlights indicate values that are close to the threshold, which do not fully exclude the compound, while red highlights indicate a failure to meet the threshold criteria.

	Goal	MoIPort-042– 626-512	MoIPort-042–633-763	RepSox	JWP00474	69811168	CDS021418
EC_50_ NEFL-NLuc assay (nM)	< 200	55	110	4000	2000	8000	50000
Efficacy (%NF-L increase)	Between 50–150%	32	38	56	35	20	38
Therapeutic Index	> 100	90	73	>25	25	12.5	> 2
NF-L ELISA	Confirms NEFL-NLuc assay	Confirmed	Confirmed	Confirmed	Confirmed	Confirmed	Confirmed
IC_50_ of p-SMAD3 (μM)	> 2 μM when possible	>100	> 100	0.025	> 100	> 100	0.015
Functional Efficacy (MEA assay)	> 80%	0	82%	100%	0	20%	0%
Molecular weight (g/mol)	≤ 500	225	210	287	195	195	267
logP	< 5	0.51	1.34	2.97	2.08	2.08	2.58
Plasma protein bound (%)	< 90	81.2	74.7	65.8	85.1	90.9	79.5
Human liver microsome CLint (μL/min/mg)	< 47	< 23.1	< 23.1	26.9	114	25.8	23.5
Intestinal permeability (bidirectional Caco-2)	P_app_ > 3x10^−6^ Efflux ratio < 10	1.9 × 10^−6^ ER=14	39 × 10^−6^ ER=0.8	38 × 10^−6^ ER=0.7	58 × 10^−6^ ER=0.8	56 × 10^−6^ ER=0.8	46 × 10^−6^ ER=0.5
BBB penetration potential (MDR1-MDCK)	P_app_ > 3× 10^−6^ Efflux ratio < 3	2.6 × 10^−6^ ER=3.2	34 × 10^−6^ ER=0.9	18 × 10^−6^ ER=1.0	58 × 10^−6^ ER=0.9	48 × 10^−6^ ER=0.8	23 × 10^−6^ ER=1.1
