# Supplementary material for: High-throughput screening of ALS patient iPSC-derived spinal motor neurons identifies novel compounds that increase neurofilament light chain expression
Source: SLAS Discov. Author manuscript; Available in PMC 2026 May 18. (PMC13182228; doi:10.1016/j.slasd.2026.100303)
Supplement: MMC1 [file NIHMS2158517-supplement-MMC1.docx]

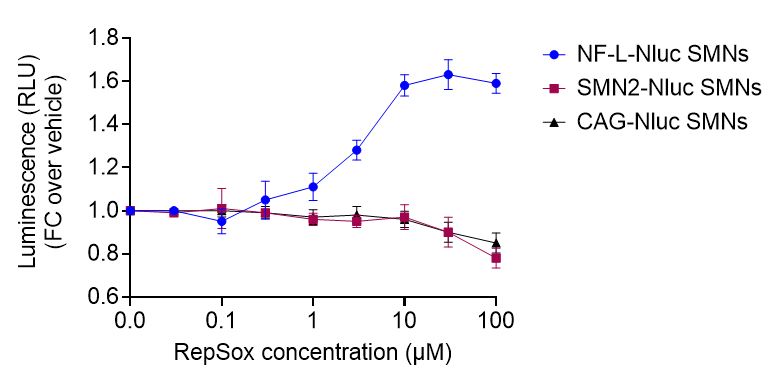


**Supplemental Figure 1.** RepSox specifically targets *NEFL* and not the *NLuc* reporter. n≥3 independent replicates for each experiment. FC: Fold change.


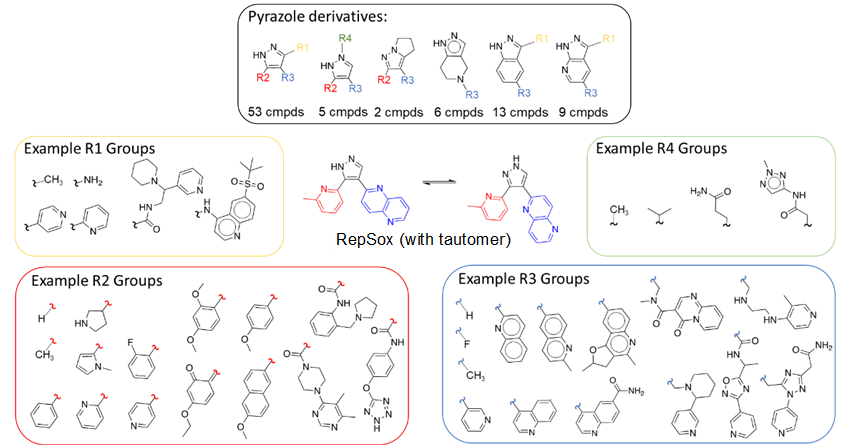


**Supplemental Figure 2.** Example commercially available RepSox analogs screened in a preliminary SAR analysis.
